# Aspergillus and Rhizopus Fungal Coinfection in a Patient With Multiple Myeloma

**DOI:** 10.7759/cureus.8050

**Published:** 2020-05-11

**Authors:** Anum Aqsa, Sami Droubi, Allison Glaser

**Affiliations:** 1 Internal Medicine, Staten Island University Hospital, Staten Island, USA; 2 Internal Medicine, Northwell Health-Staten Island University Hospital, Staten Island, USA

**Keywords:** aspergillus, rhizopus, multiple myeloma, influenza a

## Abstract

Opportunistic fungal infections are rare but life-threatening in immunocompromised patients. We discuss a case of an immunocompromised patient with multiple myeloma who presented with shortness of breath, fever, ocular palsy, and hemiplegia. She was found to have influenza A respiratory tract infection complicated by invasive aspergillosis and mucormycosis. Investigation revealed invasive fungal sinusitis and cerebritis. Serum biomarkers, beta-d-glucan, and galactomannan failed to detect fungal disease. We believe that our case is unique as there is limited data available regarding the occurrence of invasive fungal infections after Influenza infections. Furthermore, it highlights the hurdles in the diagnosis of disseminated fungal infection.

## Introduction

Invasive fungal coinfections pose a significant healthcare problem in immunocompromised patients. Host immunity and numerous physiological factors come into play to determine the spectrum of clinical manifestations. Risk factors for invasive fungal infections include neutropenia (less than 500 neutrophils/ml for at least 10 days), hematologic neoplasms, bone marrow, and solid organ transplantation, prolonged (>4 weeks) corticosteroid use, prolonged intensive care units (>21 days) stay, human immunodeficiency virus (HIV), poorly controlled diabetes mellitus and malnutrition [1].

Fungal infections are mostly transmitted by inhalation of microspores or cutaneous contact. Aspergillus species (spp) and Candida spp are the frequently isolated fungi causing infections in immunocompromised patients [1].

Infections vary in severity from mild and superficial (e.g., dermatophytosis) to invasive, systemic infections (e.g., candidiasis, aspergillosis, mucormycosis). Biopsy of tissue and culture of clinical specimens (blood, urine, tissue, sputum, and wound) is the gold standard for diagnosis. Serum biomarkers like galactomannan and beta-d-glucan assays (fungitell) are widely used now. Invasive fungal coinfections are seen infrequently in patients with multiple myeloma (MM). MM is a malignancy of plasma cells, and it increases susceptibility to many infections due to abnormalities of T cells, B cells, dendritic cells, and natural killer cells.

## Case presentation

**Figure 1:**
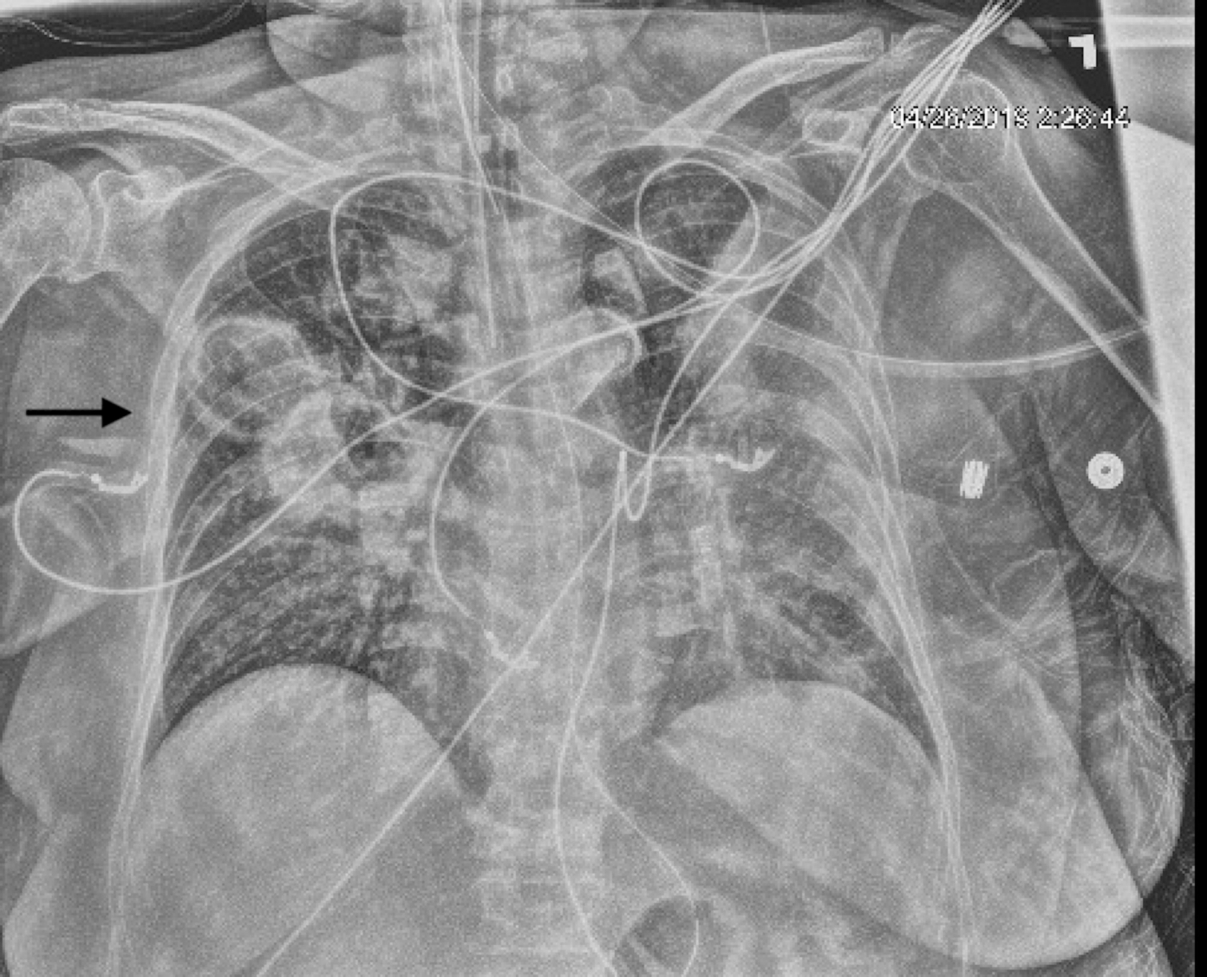
Chest X-ray showing right upper lobe cavitary lesion with a surrounding thick wall

A 77-year-old female known to have diabetes mellitus type 2, drug reaction with eosinophilia and systemic response syndrome (DRESS), and immunoglobulin G (IgG) lambda multiple myeloma presented with sudden and progressive shortness of breath for one day after her second cycle of chemotherapy. She also had associated productive cough, fever, and chills. Chest X-ray revealed new-onset bilateral parenchymal opacities. The respiratory viral panel was positive for influenza A. Blood cultures grew pan-sensitive Klebsiella pneumoniae. The patient received oseltamivir, intravenous cefepime one gram eight-hourly, and linezolid 600 milligrams 12-hourly. She was then intubated for acute hypoxic respiratory failure and started on vasopressors. Repeat chest X-ray on the fourth day of intubation showed a new right upper lobe cavitary lesion with a surrounding thick wall (Figure 1).

**Figure 2:**
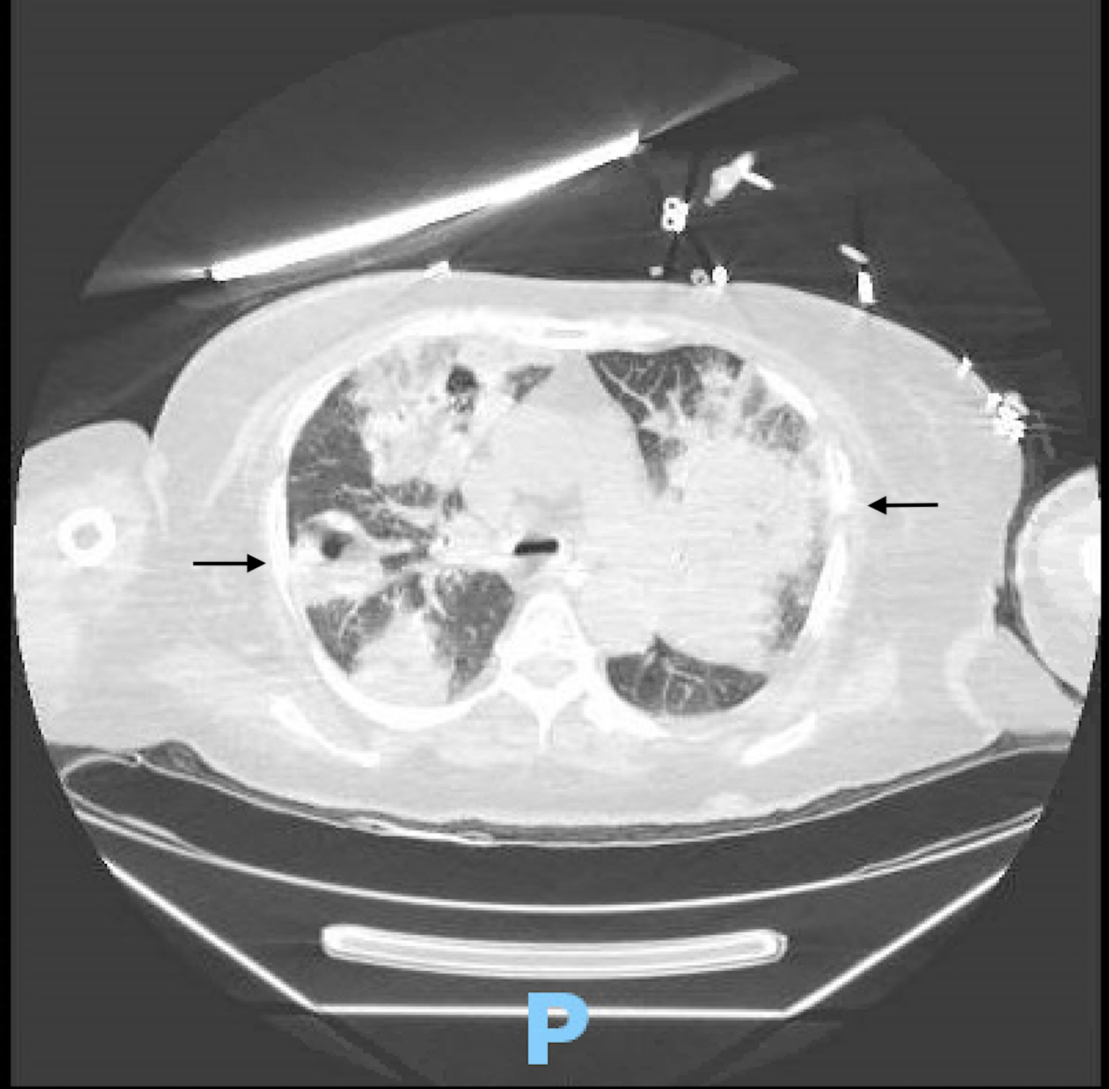
Chest computed tomography with intravenous contrast showed multifocal consolidations with central ground-glass opacities and cavitation, concerning for angioinvasive aspergillosis

Serum beta-D-glucan, serum Aspergillus galactomannan, Strongyloides antibody, and Cryptococcus antigen were negative. Bronchoscopy showed purulent secretions at the carina and dusky grey mucosa of the right upper lobe. Broncho-alveolar lavage grew Methicillin-resistant Staphylococcus aureus (MRSA), Rhizopus species, Aspergillus niger and Aspergillus fumigatus. Acid-fast bacillus (AFB) cultures were negative. Chest computed tomography (CT) with intravenous contrast showed multifocal consolidations with central ground-glass opacities and cavitation, concerning angioinvasive aspergillosis (Figure 2).

The patient was started on 5 milligrams/kilogram (mg/kg) of liposomal amphotericin B.

**Figure 3:**
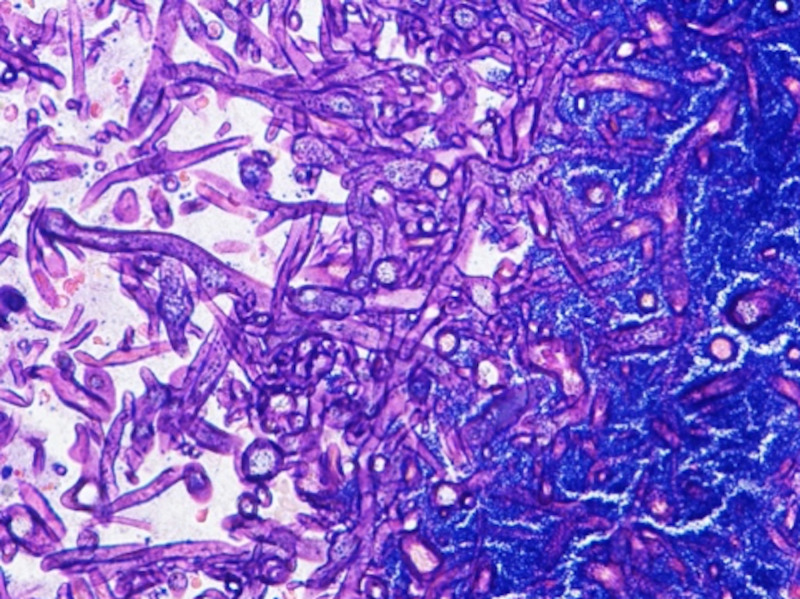
Left inferior turbinate showing thick and thin walled septate hyphae (hematoxylin and eosin stain)

**Figure 4:**
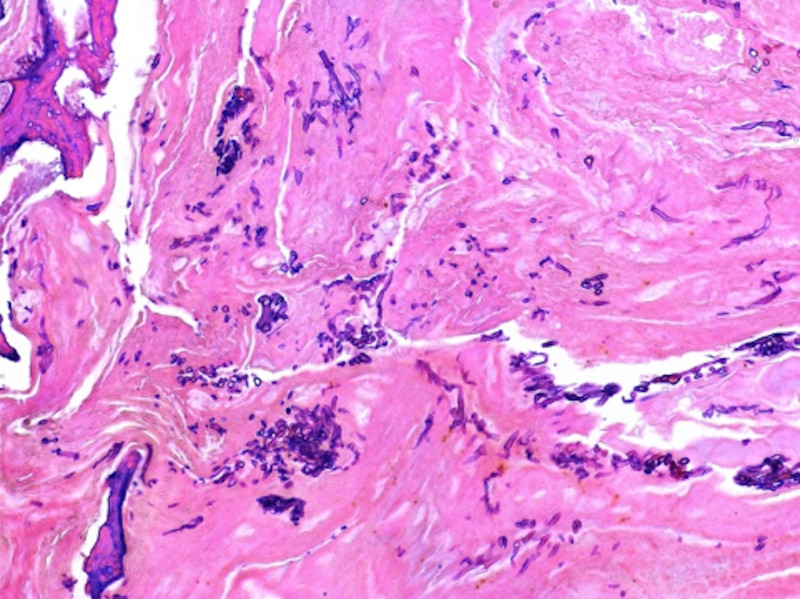
Left anterior ethmoid sinus showing cancellous bone and fibrocollagenous tissue with clusters of hyphae (hematoxylin and eosin stain)

On the fifth day of admission, she was noted to have anisocoria. Non-contrast CT head showed a new wedge-shaped area of hypo attenuation in the inferior left frontal lobe consistent with an acute/subacute infarct and partial opacification of the bilateral sphenoid, ethmoid, maxillary, and frontal sinuses consistent with pansinusitis. Emergent sphenoid sinusotomy, nasal septectomy, endoscopic total ethmoidectomy, and radical maxillary antrotomy were performed by otolaryngology. Pathology showed invasive fungal sinusitis with MRSA and Rhizopus species (Figures 3 and 4).

**Figure 5:**
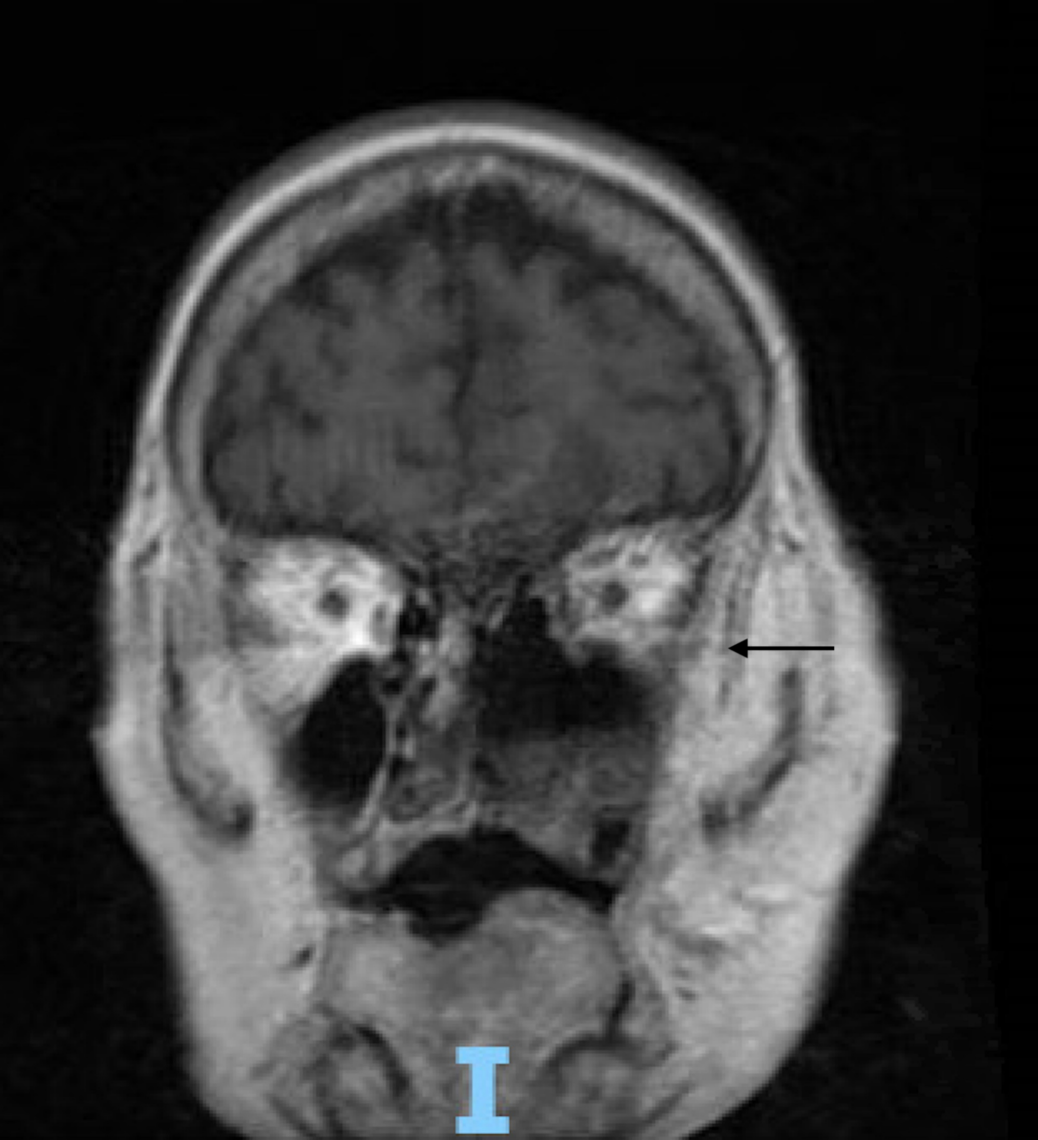
Magnetic resonance imaging of the head, orbit, face, and neck with contrast showing sinonasal postsurgical changes with residual diffuse inflammatory changes, and air-fluid levels consistent with the patient's known invasive fungal sinusitis

**Figure 6:**
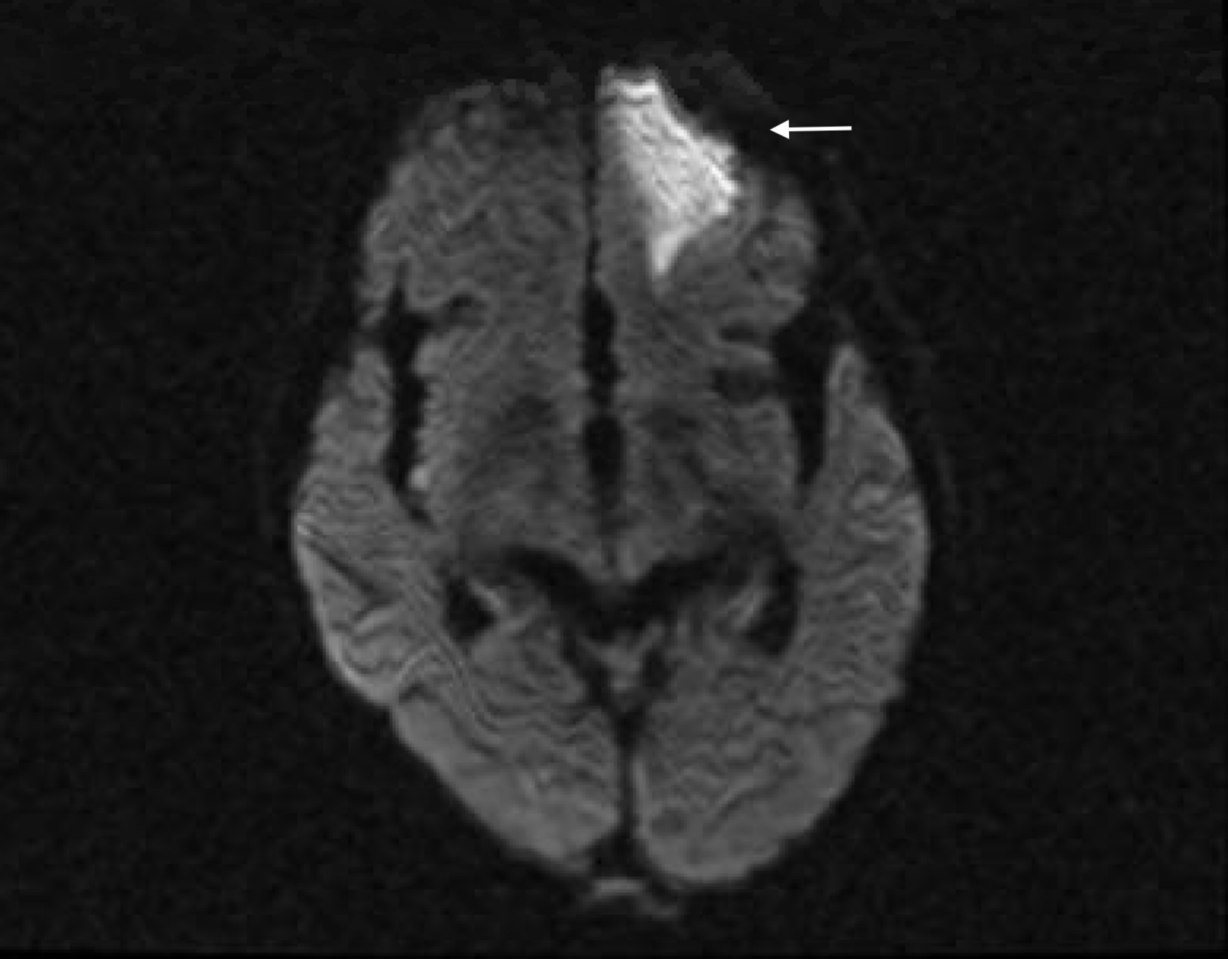
Magnetic resonance imaging of the head, orbit, face, and neck with contrast showing left frontal lobe wedge-shaped signal abnormality with restricted diffusion and mild marginal enhancement most compatible with cerebritis

**Figure 7:**
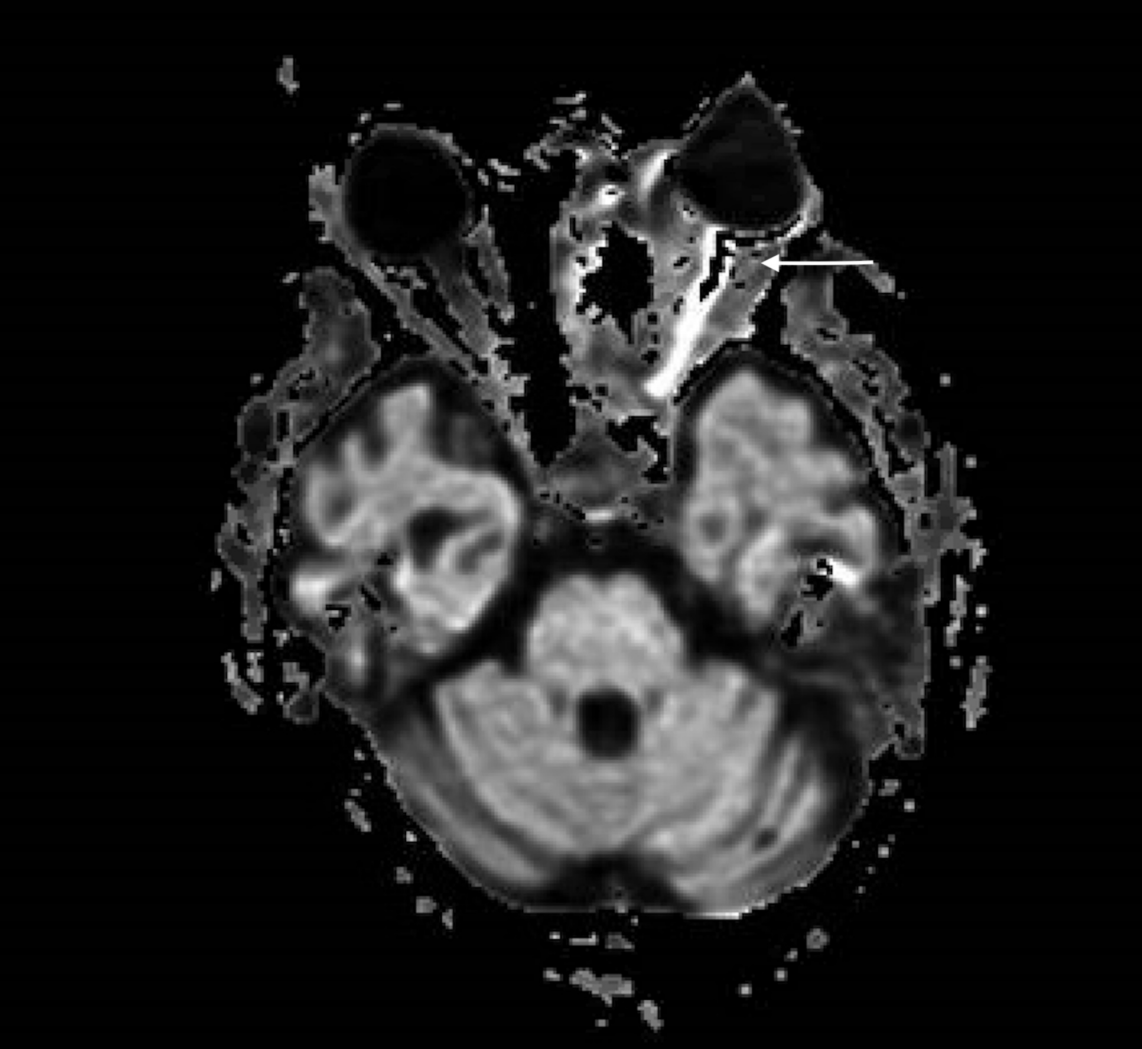
Magnetic resonance imaging of the head, orbit, face, and neck with contrast showing optic nerve inflammation/ ischemia.

Follow-up magnetic resonance imaging (MRI) of the head, orbit, face, and neck with contrast showed sinonasal postsurgical changes with residual diffuse inflammatory changes, and air-fluid levels consistent with the patient's known invasive fungal sinusitis, left frontal lobe wedge-shaped signal abnormality with restricted diffusion and mild marginal enhancement most compatible with cerebritis, and optic nerve inflammation/ ischemia (Figures 5-7).

There was also compression of the left orbital apex and cavernous sinus. After discussing the grave prognosis with the family, the patient was taken off mechanical ventilation and eventually expired.

## Discussion

Multiple myeloma is one of the most common primary bone cancers among the elderly. It increases susceptibility to many infectious diseases through B-cell, T-cell, dendritic cell and natural killer cell dysfunction, steroid use, and neutropenia from chemotherapy or marrow infiltration, impaired mucosal immunity and indwelling catheters [1, 2]. The possibility of infection is high earlier in the course of disease [3, 4]. Therefore, efforts to prevent or mitigate infection is essential to improving survival outcomes. 

Common fungi leading to infections in immunocompromised patients are Aspergillus spp, Candida spp, Cryptococcus spp, Fusarium spp, Zygomycete app, and Dematiaceous app [1]. Fungal infections were typically found during the period of progressive disease (85.7%), with patients usually having undergone a median of five cycles of therapy [5]. This finding suggests cumulative exposure to immunosuppressive agents and disease burden are more important determinants of risk for invasive fungal infections than the choice of individual therapy.

Zygomycosis, an uncommon but very severe disease, belongs to order Mucorales (class Zygomycetes). Causative organisms are identified as Mucor, Rhizopus, Absidia, and Rhizomucor species. These rapidly growing organisms are typically found on decaying vegetations and in the soil. Infection with mucormycosis often hints towards an underlying immune deficiency. Primary disease is initiated in the respiratory tract and manifests as respiratory tract infection, sinusitis, or rhinocerebral mucormycosis. These fungi rapidly infarct blood vessels, causing necrosis of surrounding tissue. Vascular occlusion also produces an acidic environment in the tissue ideal for fungal growth and protects fungi from the antifungal agents. Treatment includes early diagnosis, intravenous antifungals, control of underlying conditions, and radical surgical debridement. With intracerebral involvement, the prognosis is dismal.

Aspergillosis can manifest as either invasive aspergillosis, pulmonary aspergilloma, and allergic bronchopulmonary aspergillosis [6]. Invasive aspergillosis occurs in patients with hematological neoplasms, mainly during prolonged periods of neutropenia, but also with solid tumors, HIV/AIDS, and after an allogeneic stem cell or solid organ transplantation [7]. The common primary site of infection is lungs, followed by the central nervous system.

Invasive fungal infections usually have a nonspecific insidious disease course. Gold standard testing involves histopathological demonstration via tissue biopsy. However, newer testing modalities, like serum biomarkers galactomannan and beta-d-glucan assays, and sputum, blood, and bronchoalveolar lavage (BAL) specimen for fungal staining and culture, are widely used now. The diagnostic value of both fungitell and galactomannan has been studied in various meta-analyses and has been shown to have high negative predictive value and specificity with low sensitivity [8]. Therefore, the results of these biomarkers should be interpreted with clinical and radiographic findings. 

The literature review showed a few cases of mixed fungal infections in immunocompromised states [9-11]. We believe our case is unique as it highlights the potential for influenza A to mask advanced invasive pulmonary aspergillosis. A review of the literature concludes that Influenza infection should not be labeled as a colonizer or contaminant, especially in immunocompromised patients [12-14]. Rather, it can be independently associated with the development of invasive fungal infections. This association could be explained by the breakdown of mucosal immunity, mucociliary clearance, and secretion of interleukins. However, more studies are needed to establish causality.

## Conclusions

In conclusion, enhanced surveillance for invasive mycoses, early diagnosis, and treatment may improve clinical outcomes. More studies are needed to evaluate the applicability of diagnostic serum fungal biomarkers in such individuals. Furthermore, a deeper understanding of the association between influenza and fungal infections may help better manage these co-infections.
